# Calculating census tract-based life expectancy in New York state: a generalizable approach

**DOI:** 10.1186/s12963-018-0159-3

**Published:** 2018-01-26

**Authors:** Thomas O. Talbot, Douglas H. Done, Gwen D. Babcock

**Affiliations:** 1 Department of Epidemiology and Biostatistics, University of Albany School of Public Health, Rensselaer, NY USA; 2 Bureau of Environmental and Occupational Epidemiology, New York State Department of Health, Albany, NY USA

**Keywords:** life expectancy, geographic aggregation, spatial aggregation, health disparities, geocoding, small area analysis

## Abstract

**Background:**

Life expectancy at birth (LE) has been calculated for states and counties. LE estimates at these levels mask health disparities in local communities. There are no nationwide estimates at the sub-county level. We present a stepwise approach for calculating LE using census tracts in New York state to identify health disparities.

**Methods:**

Our study included 2751 census tracts in New York state, but excluded New York City. We used population data from the 2010 United States Census and 2008–2010 mortality data from the state health department. Tracts were assigned to 99.97% of the deaths. We removed tracts which had a majority of people living in group quarters. Deaths in these tracts are often recorded elsewhere. Of the remaining 2679 tracts, 6.6% of the tracts had standard errors ≥ 2 years. A geographic aggregation tool was used to aggregate tracts with fewer than 60 deaths, and then aggregate areas that had standard errors of ≥ 2 years.

**Results:**

Aggregation resulted in a 9.9% reduction in the number of areas. Tracts with < 2% of population living below the poverty level had a LE of 82.8 years, while tracts with a poverty level ≥ 25% had a LE of 75.5. We observed differences in LE in border areas, of up to 10.4 years, when excluding or including deaths of study area residents that occurred outside the study area. The range and standard deviation at the county level (77.5–82.8, SD = 1.2 years) were smaller than our final sub-county areas (64.7–92.0, SD = 3.3 years). The correlation between LE and poverty were similar and statistically significant (*p* < 0.0001) at the county (*r* = − 0.58) and sub-county level (r = − 0.58). The correlations between LE and percent African-American at the county level were (*r* = 0.11, *p* = 0.43) and at the sub-county level (*r* = − 0.25, *p* < 0.0001).

**Conclusion:**

The proposed approach for geocoding and aggregation of mortality and population data provides a solution for health departments to produce stable empirically-derived LE estimates using data coded to the tract. Reliable estimates within sub-county areas are needed to aid public health officials in focusing preventive health programs in areas where health disparities would be masked by county level estimates.

## Background

Life expectancy (LE) at birth is often used as an indicator of the overall health of a population. This indicator is well defined and well understood and summarizes mortality patterns across all age groups. Life expectancy at birth is an estimation of how long a newborn can expect to live, based on the length of the lives of the people who were born before them. Life expectancy has been used as an indicator to highlight health disparities between counties across the United States. Kulkarni et al. reported that life expectancy at birth ranged from 65.9 to 81.1 for males and 73.5 to 86.0 years for females across US counties [1]. Life expectancy estimates are useful at this scale for identifying health disparities across the country, but counties often have large populations and there can be significant differences in health within a county. Over three quarters of the US population live in counties with populations over 100,000.

Large differences in sub-county LE estimates have been shown for several US cities and counties. They include Baltimore, MD; New York City (NYC), NY; Alameda County, CA; Los Angeles County, CA; King County, WA; Orleans Parish, LA; Richmond, VA; and Chicago, IL [2–9]. However, sub-county LE expectancy estimates have not routinely been calculated for entire states or regions of the country.

Currently, there are efforts to promote the systematic production of sub-county estimates of LE in the US. The US Centers for Disease Control and Prevention (CDC) and the Council of State and Territorial Epidemiologist have worked with state and local health departments on the Sub-County Assessment of Life Expectancy (SCALE) Project to evaluate, develop, and adopt methods to produce LE estimates for small areas [10]. More recently the CDC Environmental Public Health Tracking (EPHT) Program [11] established a team to investigate the feasibility of producing Nationally Consistent Data Measures (NCDMs) of LE for sub-county areas which both state health departments and CDC could display online. The National Association for Public Health Statistics and Information Systems, along with the National Center for Health Statistics, have been working with vital statistics offices to geocode, improve the timeliness of, improve the quality of, and share mortality data.

There are modeling approaches for estimating LE for small areas such hierarchal Bayes or adaptive spatial filtering models [12]. These types of models rely on the fact that rates of disease are often spatially autocorrelated and that by borrowing data from neighboring areas the uncertainty of estimates in small areas can be reduced. Other approaches can be used which rely on the relationship of health indicators with covariates such as income, education, and race to estimate health indicators for small areas. These two approaches can be combined to produce small area estimates of LE [1, 13]. In NYS, our experience indicates that the public may prefer empirically-derived health indicators for diseases, such as cancer, which are calculated only from the data of the defined areas. Indicators derived from models may be viewed by the public as “black boxes” which are difficult to interpret. As discussed in the 500 Cities project [14], modeled health indicator estimates which are based on sociodemographic characteristics are insensitive to actual mortality and cannot be used to evaluate local interventions. Spatial models do have an important place in public health surveillance to better understand the factors leading to local disparities in LE. They are particularly useful where sampling is limited, or health conditions are rare.

We selected census tracts as our sub-county geographic unit for calculating empirically derived estimates LE for several reasons. Census tracts were originally designed to have homogeneous populations with respect to socioeconomic status. They do not cross county boundaries and generally have populations between 2500 and 8000. Census tracts were initially developed to retain their geographic boundaries for long periods of time so changes in population characteristics could be compared over time [15]. Because the characteristics of neighborhoods change, census tracts often become less homogeneous over time. Census tracts can also be split or merged as population increase or decrease within the tracts. The federal government recognized the need for local expertise in originally delineating and making changes in census tract boundaries. Local committees of data users were set up to assist the Census Bureau in creating and maintaining census tracts to reflect both the Census Bureau and local community needs. According to the US Bureau of the Census, the census tract is the most widely used sub-county statistical unit [15].

State and local health departments need a well-documented and systematic approach to calculate LE. This paper presents a stepwise approach for calculating LE using census tracts in New York state to identify health disparities. However, there are several challenges to producing LE estimates at the tract level. There are a lack of mortality data accurately geocoded to a fine scale such as the census tract. In 2013 only 47% of states geocoded their mortality data [16]. There can be inconsistencies in where health departments record deaths compared to where the US Census enumerates the populations which are used in the calculation of LE. In the past, there has often been a lack of access to death certificate data for residents who died in neighboring jurisdictions. In addition, sparsely populated tracts with few deaths have LE indicators with large margins of error. Large margins of error make it difficult to distinguish real differences in LE versus differences due to chance.

We have worked with both the SCALE and EPHT Projects to evaluate and develop systematic methods to produce LE estimates using census tracts. We were also interested in linking fine scale life expectancy estimates to sociodemographic indicators which have been associated with mortality [17, 18] and to compare the associations of LE and socioeconomic factors at the sub-county level to the associations we observe at the county level.

This paper describes how we overcame the challenges in calculating LE using census tracts in NY. We present the decisions made and methods used with the hope that others can learn from our experience.

## Methods

We obtained death certificate data from the New York State (NYS) Department of Health for deaths of residents of NYS excluding NYC for the years 2008–2012. The data included date of birth, date of death, sex, age at death, and residential address. Each death was assigned to one of nineteen age groups (< 1, 1–4, 5–9, 10–14, 15–19, 20–24, 25–29, 30–34, 35–39, 40–44, 45–49, 50–54, 55–59, 60–64, 65–69, 70–74, 75–79, 80–84, 85+ years of age).

We geocoded the residential addresses to census tract using a stepwise approach. First, we used the NYS GIS Program’s Geocoding Service [19] in conjunction with ArcGIS version 10.3. We geocoded 96.9% of the street addresses to the census tract. The geocoding reference files included both accurate address point (roof top) locations and street line files for the state. In some cases, the addresses were incomplete and could not be matched to the NYS address reference files. To improve our geocoding match rates, the death certificate data were next linked to hospital records to obtain more complete or accurate address information. This allowed us to geocode another 0.6% of the addresses. Using MapInfo version 12, we next determined if any of the unmatched records were in ZIP codes which were completely contained within a census tract. If so, we assigned the death to a census tract. This allowed us to geocode another 0.2%. In some cases, the address was not geocoded to the census tract because the street number was either missing or not entered correctly. We developed code in SAS version 9.4 to determine if the street was completely contained in a tract, and if so, we could assign a tract to the record without knowing the exact location of the address on the street. This allowed us to assign an additional 0.6% of the addresses to the tract.

We geocoded 98.3% of the addresses through these batch processes, but we were concerned that the remaining addresses might be clustered in individual census tracts, which would bias our LE estimates. We manually reviewed each of the remaining ungeocoded records to correct typographical errors or find residential locations not listed in the NYS street address reference files. These locations included apartment complexes and group living facilities. We used a variety of data sources and search engines to check for correct address spellings and locate residential facilities. This allowed us to geocode an additional 1.2% of the deaths. A team member, trained in interactive geocoding, evaluated addresses at a rate of approximately one address per minute. Approximately 127 h were spent interactively geocoding. For the remaining records, we used the ZIP code or town or county along with race, ethnicity, and age to impute the 2010 census tract following the method of Henry and Boscoe [20].

We obtained population data from the 2010 US Census by census tract [21]. The population counts were grouped into the same 19 age groups as the mortality data. In addition, we obtained counts from the census of the population living in group quarters. The US Bureau of the Census also provided population counts for seven different categories of group quarters. They include: correctional facilities for adults, juvenile facilities, nursing facilities, other health care facilities, college/university student housing, military group quarters, and other non-institutional facilities. Non-institutional facilities include, shelters, adult homes, work camps, convents, and monasteries. Death certificates of residents of group quarters may have different addresses from what the census uses. This can cause deaths in group quarters being counted in different tracts from where the census counts people. We excluded all tracts where 50% or more of the population lived in group quarters to reduce bias in the LE estimates. Estimates of poverty, income, and race were obtained from the 2008–2012 American Community Survey [22] for each census tract.

The counts of population and deaths by age group and tract were imported into the Life Expectancy Calculator created by the South East Public Health Public Observatory (SEPHO) [23]. We used this Excel workbook to calculate LE at birth for each census tract along with the standard error (SE) of LE in years. The calculator used the methods described by Chiang [24, 25]. The Chiang method is adjusted to include the variance term for the final age interval using the method described by Silcocks [26].

In calculating LE for small areas, investigators [27, 28] recommend aggregating data so the SE of the LE estimates are less than 2 years or a margin of error of ±4 at the 95% confidence level. An SE greater than two would lead to LE estimates that may no longer be meaningful due to random fluctuations in the estimates. As the SE increase it becomes more difficult to determine true differences in LE between areas. In addition, in areas where there are small populations and few numbers of deaths the LE calculator underestimates the SE [27, 28]. This can occur when there are number of zero deaths in the different age categories in a census tract. To provide less biased estimates of the LE SE and more stable estimates of LE, we used the Geographic Aggregation Tool [29] in an iterative process to merge areas which had less than 60 deaths. We set the GAT to keep the merged areas from crossing county and larger city and/or town boundaries (population ≥ 25,000) so the areas could be more easily identified by the public and be useful to local officials who might implement programs to improve the well-being of their residents in local neighborhoods. A city or town of 25,000 population typically has at least six census tracts.

**Fig. 1:**
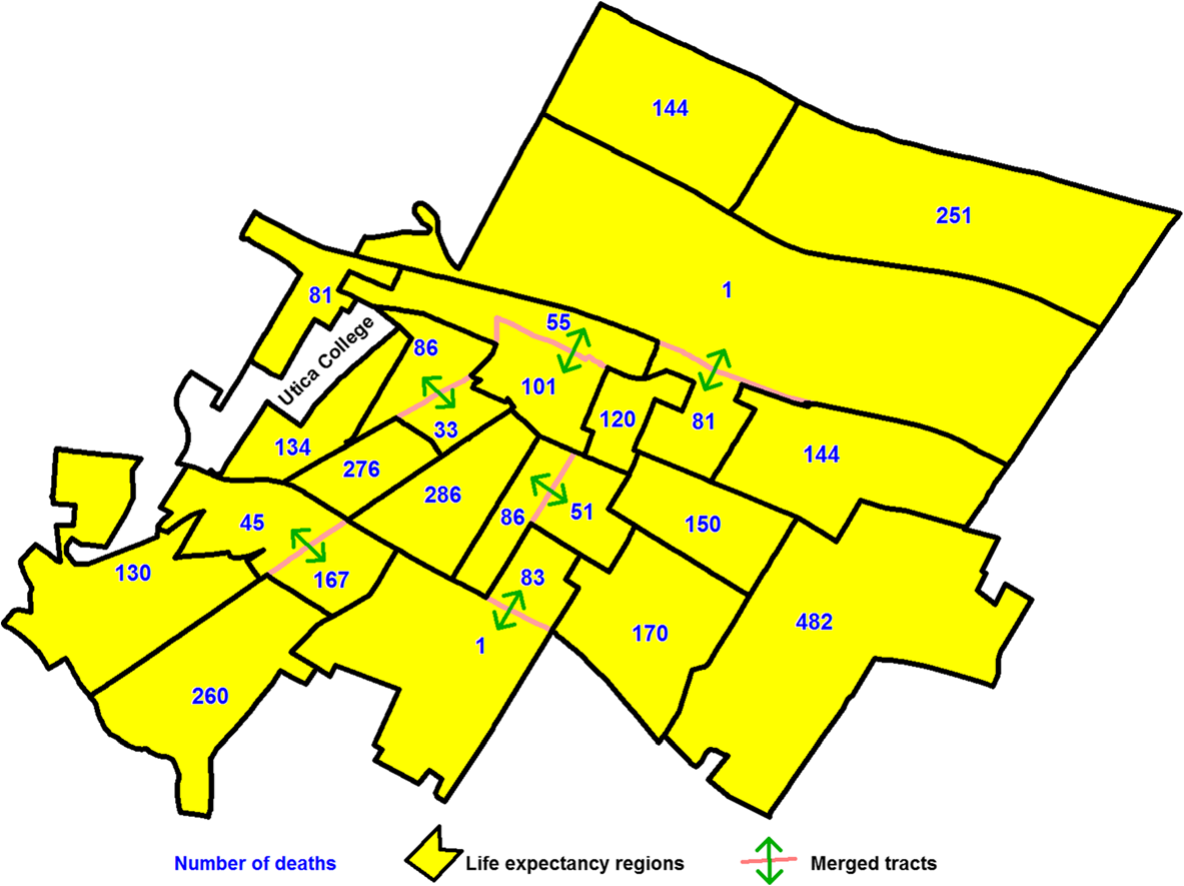
Example showing how census tracts were merged in the City of Utica, NY

The Geographic Aggregation Tool has several options for deciding how tracts are merged. We selected the option to merge the nearest tracts, rather than the tracts with fewest population or similar demographics. This option creates the most compact areas. Census tracts were first merged with neighboring tracts until at least 60 deaths were in each area. For our study population, 60 deaths occur on average in a population of 1440 over the five-year study period, i.e., 7200 person-years. After the first round of aggregation, we then calculated LE and SE for each of the areas. Areas that still had a standard error of 2 years or greater, were assigned a value of “1” to a new variable and areas that had an SE of less than 2 years were assigned a value of “2” to this new variable. We then ran areas based on this variable through the GAT again until a value greater than or equal to “2” was reached, after which we calculated life expectancy. We repeated this process until all areas had a SE of less than 2. We needed to run two iterations of these steps to ensure all areas had an SE of less than 2 years. Figure 1 shows an example of which tracts were merged in a medium size city with a population of 62,235 in upstate NY.

After aggregating census tract using the GAT we thematically mapped the LE results. We noticed very high life expectancies along our study area borders with the neighboring states and NYC. We inferred that this might be caused by deaths which occurred in health care facilities outside the study area to residents of the study area. We obtained additional death certificate reports from other states and NYC for study area residents. After adding these deaths to the sub-county areas, we compared the results of our original life expectancy estimates with the LE estimates after adding these deaths.

**Fig. 2:**
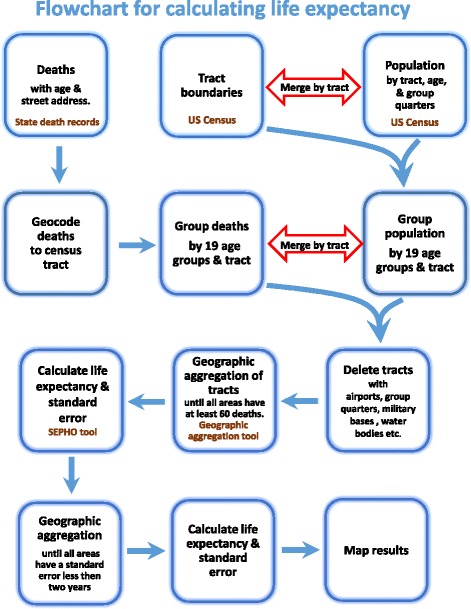
Flowchart showing the steps used in calculating sub-county life expectancy using census tracts

Next, we thematically mapped the areas by LE along with sociodemographic variables such as race and poverty. The flow diagram shown in Fig. 2 represents the steps we took to calculate LE. We also aggregated tracts based on sociodemographic characteristics of the tract rather than geographic proximity, in order to describe the relationship between LE and the sociodemographic characteristics of the study population. The Spearman’s rank correlation coefficients between LE and poverty, between LE and income, and between LE and percent of African-American population were calculated using SAS 9.4.

## Results

Our study area included all of NYS except NYC. These 57 counties included, after aggregation, 2415 sub-county areas. At the county level the percent of African-American population ranged from 0.6% to 15.1% the percent of the people living under the poverty level ranged from 5.8% to 20.0%, and the median household income ranged from $42,975 to $97,049. At the sub-county level, the percent of African-American population ranged from 0.0% to 96.1%, the percent of the people living under the poverty level ranged from 0.0% to 68.0%, and the median household income ranged from under $11,298 to over $250,000 [22].

**Fig. 3:**
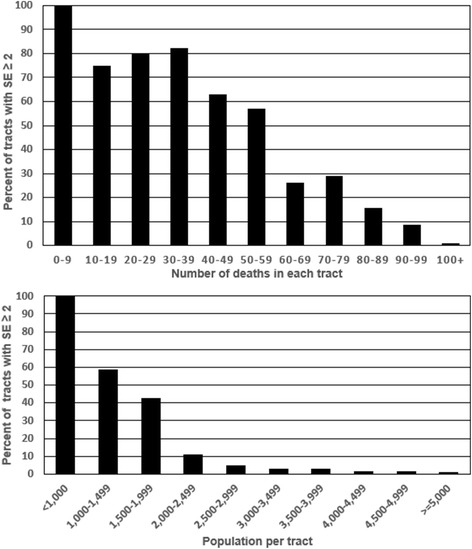
Percent of tracts with standard errors greater than or equal to 2 years by number of deaths in each tract and population in each tract

Before creating aggregated census tract areas, 6.6% of the census tracts had a SE of ≥2 years, 4.8% of the census tracts had less than 60 deaths and 8.2% of the tracts had either a SE of ≥2 years or fewer than 60 deaths. As expected, as the number of deaths and population increases in tracts, the likelihood of the tract having a SE ≥ 2 diminishes. These results are presented in Table 1 and in Fig. 3. As the number of deaths become very small, the SE estimates become unreliable. For example, SEPHO tool calculated nine of the 12 tracts which had fewer than 10 deaths with standard errors of 0.

**Table 1:** Number of tracts, number of tracts with SE ≥ 2, and percentage of tracts with SE ≥ 2 by number deaths and population

Number of deaths in tract	Number of tracts	Number of tracts with SE ≥ 2	Percent of tracts with SE ≥ 2
0–9	12	12*	100.0
10–19	4	3	75.0
20–29	10	8	80.0
30–39	17	14	82.4
40–49	27	17	63.0
50–59	58	33	56.9
60–69	73	19	26.0
70–79	79	23	29.1
80–89	114	18	15.8
90–99	116	10	8.6
100+	2169	21	1.2
Population of tract			
< 1000	17	17*	100.0
1000–1499	51	30	58.8
1500–1999	133	57	42.9
2000–2499	233	26	11.2
2500–2999	287	14	4.9
3000–3499	279	8	2.9
3500–3999	345	10	2.9
4000–4499	325	5	1.5
4500–4999	261	4	1.5
> =5000	748	7	0.9
Total			
Study area	2679	178	6.6

* Tracts with less than 10 deaths were included in this category since the SE could not be reliably calculated

Of the NYS Department of Health death certificates, of residents in the study area who also died in the study area, we assigned 99.97% of the death records to the 2010 census tract. We observed that in areas where most of the population lived in group quarters, there were many fewer deaths than expected. In some tracts, there were populations of several thousand persons with no deaths reported over the five-year study period. This occurred in tracts with correctional and higher education facilities. We also observed this effect on a large military base in upstate NY which was comprised of two tracts. We excluded these 54 tracts from further analyses, as shown in Table 2. These tracts included only 1.5% of the population and 0.47% of the deaths in our original study area. Excluding these areas increased the LE expectancy by only 9 days in the overall study area.

**Table 2:** Number of tracts, population, percent of population, number of deaths and percent of deaths excluded from study area

	# of tracts	Population (% of population)	# of Deaths (% of deaths)
Original study area	2751	11,202,969 (100.00)	473,356 (100.00)
Excluded tracts with only water area	15	0 (0.00)	0 (0.00)
Excluded with ≥50% pop. in group quarters	52	153,532 (1.37)	2121 (0.45)
Excluded military base	2	13,726 (0.12)	59 (0.01)
Excluded airports	2	4 (0.00)	0 (0.00)
Excluded unpopulated island	1	0 (0.00)	0 (0.00)
Final study area after exclusions	2679	11,035,707 (98.51)	471,176 (99.54)

After exclusions, the number of deaths was 471,176 and the population was 11,035,707 in our study area. The population of the tracts ranged from zero to 10,803. The mean population per tract was 4119. There were four tracts with no population that were included in our final study area. These were aggregated to neighboring tracts. Two of these tracts were on native American Reservations, and one was a correctional facility which bordered on another tract containing a correctional facility. The fourth tract with no population had two deaths. We verified this tract had occupied residences in the tract. We believe the US Census incorrectly failed to assign a population to this tract. Independent sources showed occupied apartment complexes in this tract. Running the 2679 tracts through the Geographic Aggregation Tool created 2415 areas. This resulted in a decline of 9.9% in the number of geographic areas. After geographic aggregation, the minimum population size increased to 1044, maximum population size increased to 25,754, and average population size increased to 4570.

**Fig. 4:**
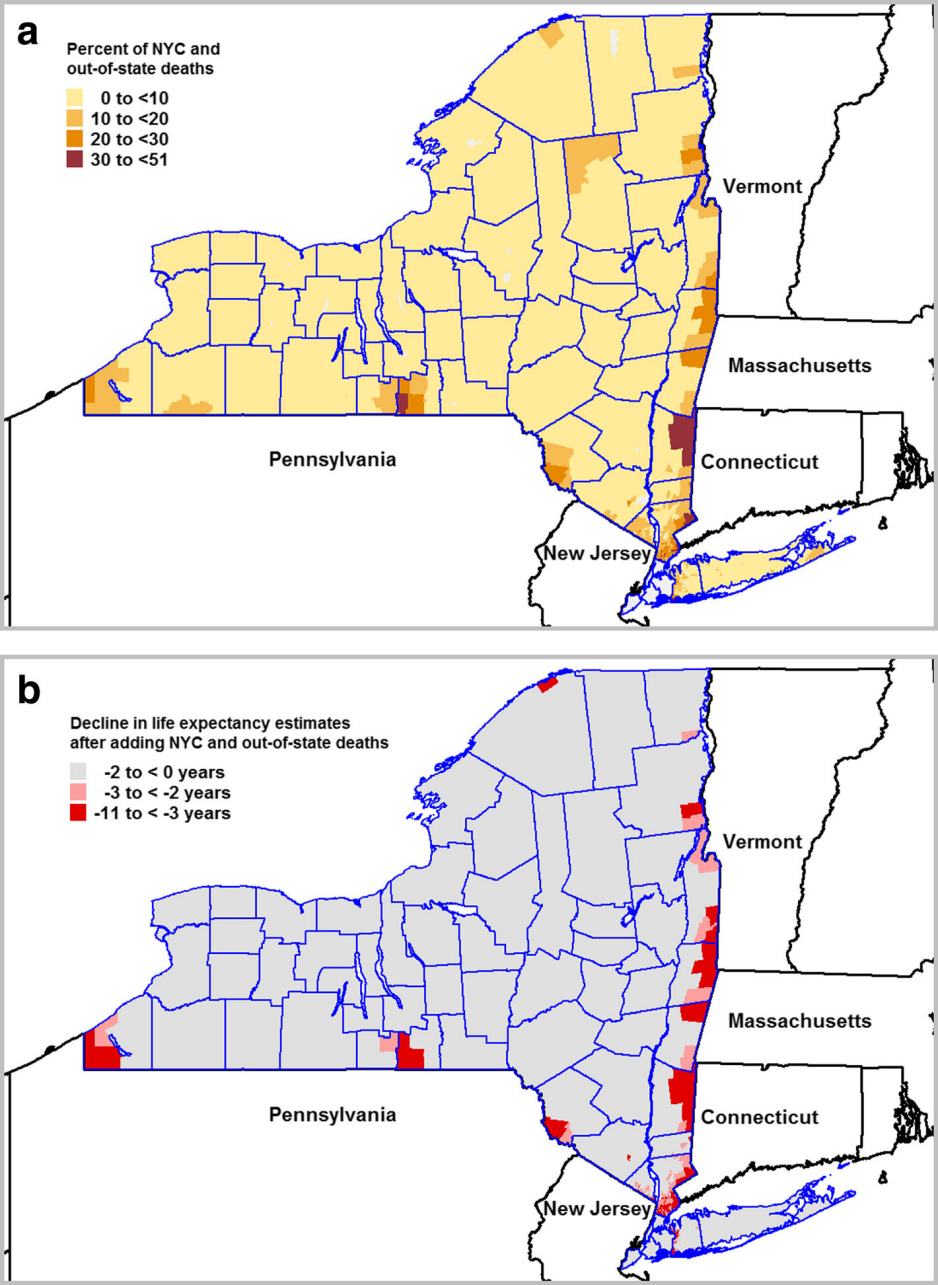
Impact of adding out-of-state and NYC deaths to local estimates of life expectancy in aggregated areas. **a** Percentage of study area residents that died in NYC or out-of-state. **b** Change in life expectancy estimates after adding NYC and out-of-state deaths

We also compared LE estimates based only on deaths in our study population which occurred in our study area, with LE estimates we calculated after adding in death records of our study population which occurred in NYC and other states. Prior to adding the NYC and out-of-state deaths, we noticed several of areas along the borders of neighboring states and NYC with very long life expectancies. We recalculated the LE in the 2415 areas after adding in 11,007 additional death records from other states and 11,371 records from NYC. After adding in these deaths, the number of deaths in our sub-county areas ranged from 62 to 1011 and the average number of deaths was 195. As shown in Fig. 4a up to 51% of the deaths in residents of border areas occurred outside the study area. Once we added the NYC and out-of-state deaths back in, we saw a decline in LE up to 10.4 years as seen in Fig. 4b.

**Fig. 5:**
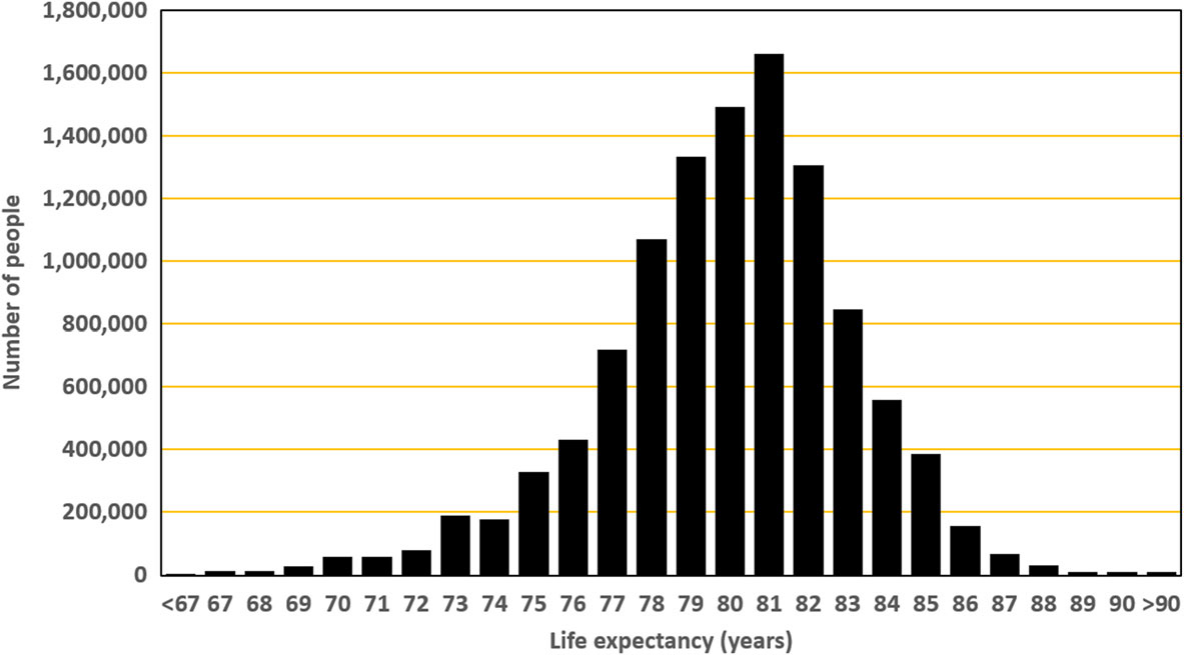
Population by life expectancy. Aggregated areas were merged based on LE, and the number of people was calculated

The LE of the study area after the area exclusions and adding out-of-state deaths and NYC deaths which occurred to residents of the study area was 80.2 years. This compares with the US life expectancy which ranged from 78.1 in 2008 to 78.8 in 2012 [30, 31]. Figure 5 shows a normal distribution of the population by LE. In the 2415 areas, the maximum LE was 92.0 years, the minimum LE was 64.7 years, and the standard deviation was 3.33 years.

**Fig. 6:**
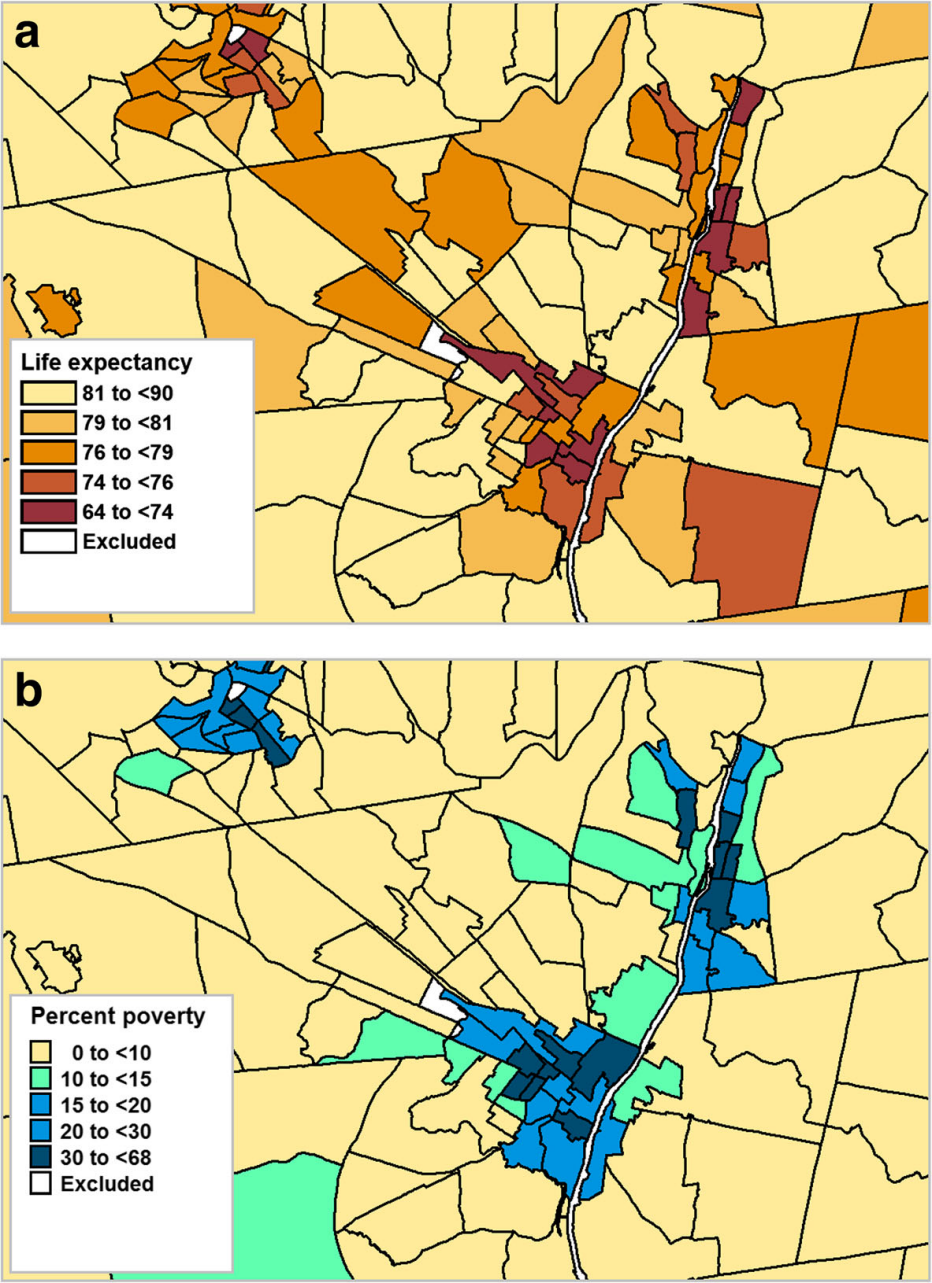
Thematic Maps of the New York State Capital District after aggregation. **a** by life expectancy. **b** by % poverty

**Fig. 7:**
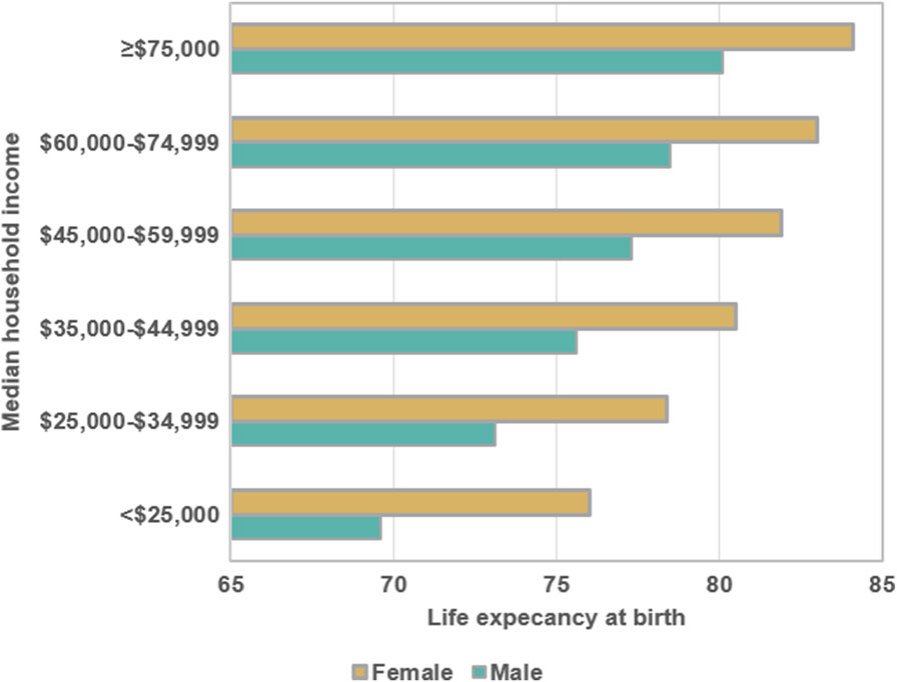
Life expectancy by median household income for males and females. Tracts were merged based on income level, and LE was calculated for merged areas

We created choropleth maps of LE in conjunction with the percent poverty, household income, and percent African-American population. Figure 6 provides an example showing the association between poverty and LE. Choropleth maps sometimes can mask the geographic relationships due to the varying sizes of the aerial units and the how the class breaks on the map are selected [32]. To view these relationships, we aggregated the census tracts based on sociodemographic variables and graphed the results. For example, Fig. 7 clearly shows the positive association between LE and household income in males and females. Use of tables also provided another option in displaying sociodemographic data in conjunction with LE estimates. Table 3 provides LE estimates after aggregating tracts based on race and poverty. From this table, we saw little difference in LE in African-American communities compared to non-African-American communities in areas with percent of population living in poverty is below 10%. However, this was not the case in the areas with the higher poverty levels (≥25%) where communities in which the majority of the population are African-American have a 4.4 year shorter LE compared to communities which contain less than 2% African-Americans.

**Table 3:** Life expectancy at birth and 95% confidence intervals (CI) by percent African-American and percent poverty in aggregated census regions

		Percent poverty			
Percent African- American	0 - < 2%	2 - < 10%	10 - < 25%	≥ 25%	Total
0 - < 2%	82.6 (±0.17)	81.4 (±0.08)	79.2 (±0.12)	78.5 (±0.42)	80.9 (±0.06)
2 - < 10%	83.4 (±0.27)	81.4 (±0.09)	79.0 (±0.13)	77.5 (±0.30)	80.7 (±0.07)
10 - < 25%	82.2 (±0.74)	80.5 (±0.22)	79.2 (±0.18)	74.7 (±0.26)	78.7 (±0.12)
25 - < 50%	*	81.4 (±0.37)	77.8 (±0.29)	75.1 (±0.28)	77.7 (±0.18)
≥ 50%	*	80.5 (±0.56)	77.5 (±0.40)	74.1 (±0.35)	76.4 (±0.24)
Total	82.8 (±0.14)	81.3 (±0.06)	79.0 (±0.07)	75.5 (±0.14)	80.2 (±0.04)

*Less than 90 deaths

We compared the ranges and standard deviations (SD) of our LE expectancy estimates, for the 57 counties and the 2415 sub-county areas in our study area. As expected both the LE range and LE SD at the county level (77.5–82.8, SD = 1.2 years) were much smaller than our sub-county areas (64.7–92.0, SD = 3.3 years). We also compared the correlations between the LE and socioeconomic variables at the county level and sub-county level. The Spearman’s Rank-order correlation between LE and poverty were found to be similar and statistically significant (*p* < 0.0001) at both county level (*r* = − 0.58,) and at the sub-county level (r = − 0.58). We obtained similar results for LE and income (*r* = 0.64 at the county level, *R* = 0.64 at the sub-county level). However, we saw different correlations between LE and percent African-American at the county level (*r* = 0.11, *p* = 0.43) than at the sub-county level (*r* = − 0.25, *p* < 0.0001).

## Discussion

We demonstrated it is feasible to geocode deaths to census tract with minimal loss of subjects. Census tracts were found to be useful building blocks for creating the areas used to produce choropleth maps of LE. We present a framework that state and local health departments can use to overcome many of the challenges in calculating LE for census tracts. Problems with unstable estimates due to small numbers can be overcome by temporally aggregating deaths over a five-year period. Geographic aggregation is used to ensure adequate numbers of deaths in sparsely populated census tracts. Geographic aggregation did not significantly degrade the geographic scale from the original census tract level. We confirmed the need to acquire death certificate data from neighboring jurisdictions to avoid biased LE estimates in border areas.

The approach we used to calculate LE using census tracts can be applied in other states since the tract population sizes in our study area are comparable to other areas of the country. The median population size of census tracts in NYS excluding NYC is 3952 which compares to 3426 in NYC. Across all US states the median population size of census tracts ranges from a minimum of 2785 in North Dakota to a maximum of 4714 in Idaho [21].

Although deaths which occur while living at colleges, military bases, or prisons are supposed to be coded to these locations [33], we observed this was not the case. We excluded these areas since the number of deaths in these areas are unknown. These tracts however only represented a small portion of our study population and we observed that excluding these tracts had little impact in the LE estimate of the overall study area. However, there may be a much larger upward bias in LE estimates in some tracts if deaths which occur among residents of these institutions are more likely to be miscoded to specific tracts. Unfortunately, we did not have the data to re-assign these deaths to the place of residence as defined by the census or assign populations to where these deaths were assigned after geocoding death records.

The data showed the range and variation of LE estimates and sociodemographic indicators was much larger at the sub-county level compared to the county level. Therefore, looking at the data at the sub-county level reveals the large disparities in socioeconomic status and life expectancy between communities within a county. For example, Nassau County, NY (population = 1,339,532) has lowest percentage of people living under the poverty level (5.8%) of any of the 57 counties in our study. Its population is 11.4% African-American, and it has a high life expectancy of 82.2 years. However, a census tract in the center of the county has a life expectancy of only 70.7 years, one of the lowest of any our sub-county areas (1st percentile). It has 15% of the population living under the poverty level, and 48% are African-American.

The authors of SEPHO Technical [27] report recommended that 5 years of mortality data be used to estimate LE at census ward in the United Kingdom. Populations of wards are comparable to US census tracts, with 99% of the tracts and wards having populations of at least 1000. They recommended at least 5000 person-years is needed to produce standard error estimates of 2 years, based on the population age structure and mortality rates in England. However, if a SE of 2 years is the average, then a large portion of the areas of this population size would have a SE above two. For tracts with populations between 1000 and 1499, we show 59% have standard errors ≥2 years (Table 1). For a population size of 1440 (7200 person-years), we expect 60 deaths on average. When we aggregated tracts to have a least 60 deaths, and then aggregated areas with a SE error of greater than or equal to 2 years, we obtained sub-county areas close to the original census tract level.

We would need to increase the spatial or temporal aggregation to provide reliable LE estimates for males and females separately. Expanding the study population through aggregation would further reduce the spatial resolution of the sub-county estimates. Expanding the study period would be difficult due changes in the age distribution of the population over time. Therefore, we chose not to calculate LE by gender for the sub-county areas.

There are several limitations in our methods. The LE estimate is only a snapshot of the population currently living in the tract, and not the measure of the environment that the local population was exposed to over a lifetime. We do not know how long people who die in a census tract lived in the tract. People in poor health may move to be closer to health care facilities, but people in good health may move to areas where they can find employment (healthy worker effect). LE estimates cannot differentiate between “healthy places” and places where healthy people migrate to (or the converse). Another limitation of the life expectancy indicator is that it is not predictive for individuals, but instead represents an aggregate health metric. The predictive ability of life expectancy is limited due to the shifting of health burdens over time in the population due to medical advances, changes in behavior and other risk factors. For example, all-cause and cardiovascular-specific mortality rates have been historically declining over the past 50 years, even as obesity- and diabetes-related complications are on the rise [34].

A third limitation is that estimating population by age group over time can be problematic. We use a point estimate of population from the 2010 to estimate the population for our entire five-year study period, so if there were large changes in the population age profile in an area over this period it may also bias the result. We preferred using the US Census estimates which is based on a 100% count of the population as opposed to using the American Community Survey (ACS) which only sampled one in 12 occupied housing units in NYS during our study period. The margin of error of ACS estimates by age group are often large for census tracts in this survey. The ACS also does not provide the population of children under 1 year of age separately from the 1–4 year age group. The SEPHO Calculator requires an estimate of the < 1 age group to account for the effects of infant mortality when constructing an abridged life table. However, it would be feasible to use birth records to estimate the population of infants in a census tract. Linear interpolation can also be used to estimate populations in the intercensal years, but we would need to wait for the next release of the 2020 decennial census to use this approach.

Lastly, estimating the true uncertainty of the LE estimates is difficult. In addition to the stochastic error related to small numbers, error is introduced when death certificates are recorded, populations are enumerated, and deaths are geocoded. The SEPHO LE calculator estimates the standard error based solely on the population and number of deaths placed into each age category. The calculator does not consider the propagation of errors from our input files and geocoding processes. As we improve the methods and quality of the input data, errors are reduced. For example, over the years, we have seen a reduction in the positional error in geocoding as we moved from methods which estimate the location of an address through the interpolation of a street number along a street, to placement of the house at the centroid of a property [35], to the assignment of the address based on the rooftop of the house [19]. We are also seeing improved sharing of death certificate data from neighboring jurisdictions. Therefore, future LE estimates may be more accurate than current ones.

## Conclusion

Over the past several years there have been advances in reference files used for geocoding, the development of easy to use tools for calculating LE [23] and for aggregating areas with small numbers [29] which now make it feasible to create estimates of LE for sub-county areas across the country.

The study area included over 2600 census tracts. States and other large jurisdictions can easily aggregate areas to create stable LE estimates using the Geographic Aggregation Tool. However, smaller jurisdictions such as cities should consider using local knowledge of their neighborhoods to create areas for reporting health indicators. An example of local health reporting districts can be found on the NYC Department of Health and Mental Hygiene web page [3] in which the city health department created health profiles for 59 community districts which include a variety of health indicators including the leading causes of death and LE. Each district is made from an aggregation of census tracts. The populations of these districts ranged from 52,607 to 255,707, which is much larger than our sub-county areas.

Although both the EPHT and SCALE LE teams were formed to investigate creating sub-county estimates of LE across the US, this might not be feasible in all areas. According to the 2010 Census there were 163 counties located across 24 states which have populations of less than 3000 people. In these areas, producing empirically derived stable sub-county estimates is not practical. For example, if we were to break a county of 3000 people into two areas with 1500 people in each area, we see from Table 1 that there is about a 50% likelihood that each of these areas would have an SE ≥2 years after aggregating the deaths over 5 years. In addition, a number of counties with small populations may only be composed of one census tract since the average size of a census tract is around 4000 people.

The framework for calculating LE using census tract data is unique from previous approaches for calculating LE for small areas in that we: 1) coded all our deaths to the census tract, 2) identified areas where LE may not be reliable due to border effects or population living in group quarters, and 3) ensured all small-area LE estimates had a SE of less than 2 years.

Maps of LE can be used to identify areas with short life expectancies so that public health actions can be taken. They can be used to catch the attention of the public and news media, which can provide an opportunity to deliver public health messages.

## Abbreviations

EPHT: Environmental Public Health Tracking
LE: Life expectancy
NY: New York
NYC: New York City
NYS: New York state
SCALE: Sub-county Assessment of Life Expectancy
SD: Standard deviation
SE: Standard error
SEPHO: South East Public Health Public Observatory
US: United States
